# The impact of socio-economic status on outcomes after unplanned intensive care unit admissions in Australia: a retrospective observational cohort study

**DOI:** 10.62675/2965-2774.20260417

**Published:** 2026-07-16

**Authors:** Sing Chee Tan, Daniel Capurro, David Pilcher

**Affiliations:** 1 Northern Hospital Epping - Epping Department of Intensive Care Medicine Epping Austria Department of Intensive Care Medicine, Northern Hospital Epping - Epping, Victoria, Austria.; 2 University of Melbourne Centre For Digital Transformation of Health Melbourne Australia Centre For Digital Transformation of Health, University of Melbourne - Melbourne, Victoria, Australia.; 3 Alfred Hospital Department of Intensive Care Melbourne Australia Department of Intensive Care, Alfred Hospital - Melbourne, Victoria, Australia.

**Keywords:** Socioeconomic factors, Intensive care units, Hospital mortality, Health status disparities, Universal health coverage, Australia

## Abstract

**Objective:**

To examine the association between socioeconomic status and patient outcomes following unplanned intensive care unit admissions in Australia, using national data linked across public databases.

**Methods:**

We conducted a national retrospective cohort study of all adult unplanned intensive care unit admissions in Australia between January 2017 and December 2019, using data from the Australian and New Zealand Intensive Care Society Adult Patient Database. Socioeconomic status was determined using the Australian Bureau of Statistics’ 2016 Index of Relative Socio-Economic Advantage and Disadvantage (IRSAD), linked by patient postcode. The primary outcome was hospital mortality, adjusted for severity of illness, remoteness, year of admission, and intensive care unit, using a mixed-effects logistic regression model.

**Results:**

A total of 245,867 intensive care unit admissions were included. Patients in the most disadvantaged quartile were less likely to be treated in tertiary or private hospitals. Still, there was otherwise no significant difference in demographic profile, intensive care unit interventions received, or reasons for admission. In the multivariate analysis, there were no significant differences in adjusted hospital mortality across IRSAD deciles (p = 0.3).

**Discussion:**

In this national study, socioeconomic status was not associated with hospital mortality after adjustment for illness severity and hospital factors. These findings suggest that structural health system features, such as universal access to critical care in Australia, may mitigate the adverse effects of socioeconomic disadvantage among unplanned intensive care unit outcomes observed in other health systems. Further research is warranted to explore the pathways linking socioeconomic status, health access, and intensive care unit admission characteristics.

## INTRODUCTION

Being from a lower socioeconomic status (SES) background has been demonstrated to be associated with worse health outcomes across a range of measures, ranging from mental health to chronic disease outcomes.^(1-4)^ The impact of socioeconomic disadvantage on intensive care unit (ICU) outcomes remains less clearly defined. In particular, much of the existing literature on this topic originates from high-income countries, where health systems and access to funding vary considerably. An international systematic review suggested an association between lower SES and adverse ICU outcomes,^(5)^ yet individual studies conducted predominantly in North America and Europe have reported heterogeneous and sometimes conflicting results.^(6-8)^

One plausible reason for this variation is jurisdictional differences in healthcare delivery, particularly the extent to which critical care delivery is mediated by insurance status or out-of-pocket costs. In Australia, public hospital care is provided at no direct cost to patients and is jointly funded by the state and federal governments. This ensures that access to critical care is determined by clinical need rather than ability to pay. The availability of government-subsidized critical care, as in Australia, may ameliorate some of the drivers of outcome inequality observed in other settings.^(9,10)^ This underscores the importance of assessing the impact of SES on ICU outcomes in the Australian context, rather than extrapolating from international studies conducted in different settings.

To date, this has not been examined at the national level in Australia and has been limited to single-centered or jurisdictional studies.^(11,12)^ This study aims to assess the impact of socioeconomic status on the short-term mortality of patients with an unplanned admission to Australian ICUs. We hypothesize that lower SES status will be associated with worse outcomes.

## METHODS

### Study design and setting

This was a national, multicentre, retrospective cohort study examining all adult patients with unplanned ICU admissions in Australia and reported according to the STROBE reporting guidelines for observational cohort studies. Data from 1 January 2017 to 31 December 2019 were used to avoid the confounding effect of the coronavirus disease 2019 (COVID-19) pandemic.^(13)^

### Participants

Patients were included if they were aged 16 years or older and were an unplanned admission to an adult ICU in Australia between 1 January 2017 and 31 December 2019. Unplanned admissions are defined as patients admitted to the ICU on an emergency basis or those admitted following surgical procedures. Patients were excluded if they were admitted for palliative care or organ donation, were an ICU readmission, or were missing primary outcome data. Patients who were transferred to or from another ICU were excluded because it was not possible to link illness severity measures and outcomes across ICU episodes recorded at different sites. Postcodes were excluded if they did not have an associated Index of Relative Socio-Economic Advantage and Disadvantage (IRSAD) score.

Patient data and IRSAD deciles were linked using patient-specific postcodes. Patients without valid postcodes captured were excluded.

### Data sources

Patient data were derived from the Australian and New Zealand Adult Patient Database (ANZICS APD). This binational registry contains de-identified information on more than 95% of ICU admissions in Australia. Data comprises demographic, clinical information, and physiological parameters from the first 24 hours of ICU admission, as well as the severity of illness based on mortality predictions by the Australian and New Zealand Risk of Death (ANZROD) calculation.^(14)^

Socioeconomic data were derived from the postcode-specific 2016 IRSAD, available from the Australian Bureau of Statistics. This accounted for multiple components of SES, such as income, education, and employment, and provided an overall score across a postcode, averaging out pockets of relative advantage and disadvantage. These scores were reported as deciles, with 1 being the most disadvantaged and 10 being the most advantaged.^(15)^ Geographical remoteness was included as an adjustor, using the postcode-based 2017 remoteness areas classes, derived from the Accessibility and Remoteness Index of Australia.^(16)^

### Variables and statistical analysis

The primary outcome was hospital mortality presented as an odds ratio for each decile, referenced against the most advantaged IRSAD decile of 10. Secondary outcomes were unadjusted ICU mortality, hospital length of stay, ICU length of stay, and post-hospitalization destination. Exploratory data analysis was performed by dividing IRSAD groups into quartiles. Continuous variables were reported as mean with standard deviation and/or median with interquartile range, and categorical variables were reported as proportions. Comparisons between groups were performed with tests appropriate to the data distribution. Baseline characteristics were compared using standardized mean differences; values < 0.1 indicated good balance.

Adjusted mortality for each IRSAD decile was assessed using a mixed-effects multivariate logistic regression model, adjusted for illness severity (ANZROD), remoteness class, and year of admission, with individual ICUs modeled as random effects. Hospital type was excluded from the multivariate modelling due to collinearity concerns, but retained descriptively. This method was adopted as a method of risk adjustment to account for the hierarchical clustering of outcomes related to patients being treated by similar teams of clinicians at each ICU. Covariates were selected a priori based on validated predictors of outcome.^(14)^ A likelihood ratio test was used to assess across-group differences for SES deciles.

All analyses were done in R for Windows (Build 492), using the tidyverse package for data wrangling and the lme4, car, and broom packages. mixed packages for mixed-effects modeling.

Ethics approval was obtained from the Northern Hospital Research Ethics Committee (QI49.2022), and the ANZICS CORE Management Committee granted data access. Consent was not required as only routinely collected deidentified data was used.

## RESULTS

### Study flow

**Figure 1:**
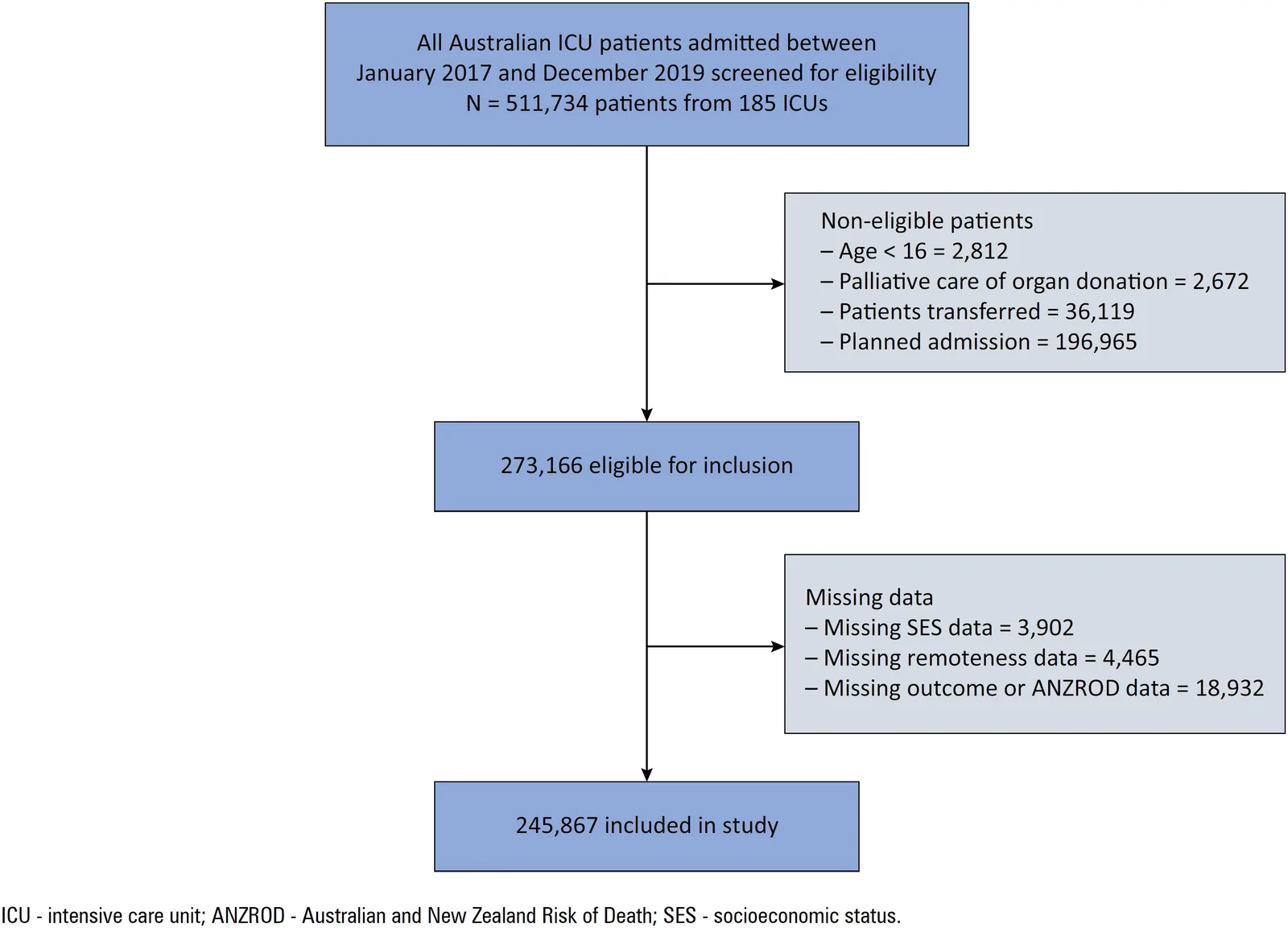
Study flow diagram.

There were 511,734 Australian ICU admissions identified from the ANZICS APD within the study period, of which 273,166 admissions were eligible for inclusion in the study. A further 18,932 admissions had missing outcome or risk adjustment data, and 3,902 of these lacked a valid postcode. 2,631 postcodes were extracted from the Australian Bureau of Statistics dataset, of which 1 did not have an IRSAD score. After applying exclusion criteria, 245,867 ICU admissions were included for the final analysis (Figure 1).

Planned admissions were defined as planned or foreseen admissions to the intensive care unit following elective surgery, or a non-emergency direct admission.

### Patient characteristics

Most patients were male with a median age of 64.7 years. Most admissions were in tertiary hospitals (43.4%), followed by metropolitan hospitals (21.1%), rural or regional hospitals (21.3%), and, lastly, private hospitals (14.2%). Most admissions were for respiratory conditions, followed by gastrointestinal and cardiac conditions. Other patient characteristics are listed in table 1.

**Table 1:** Baseline characteristics of adult patients with unplanned intensive care unit admissions in Australia (2017 - 2019), stratified by socioeconomic status according to IRSAD decile groups

		Overall	IRSAD deciles				Standardized mean difference
			1 - 3	4 - 5	6 - 8	9 - 10	
Total number		245,867	50,011	73,913	65,996	55,947	
Sex (%)							0.011
	Female	111,310 (45.3)	22418 (44.8)	33,730 (45.6)	29,677 (45.0)	25485 (45.6)	
	Male	134,435 (54.7)	27573 (55.1)	40,145 (54.3)	36,287 (55.0)	30,430 (54.4)	
	Other/Indeterminate	122 (0.0)	20 (0.0)	38 (0.1)	32 (0.0)	32 (0.1)	
Age		64.70 [48.41 - 75.90]	64.00 [48.83 - 74.94]	64.63 [48.40 -75.57]	64.02 [47.60 -75.37]	66.40 [49.00 - 77.80]	0.05
ICU source of admission							0.064
	Operating theatre/recovery	69,076 (28.1)	13,790 (27.6)	19,945 (27.0)	18,925 (28.7)	16,416 (29.3)	
	Emergency Department	118,422 (48.2)	25,025 (50.0)	37,202 (50.3)	30,579 (46.3)	25,616 (45.8)	
	Ward/CCU/Other HDU	56,983 (23.2)	10,961 (21.9)	16,390 (22.2)	16,038 (24.3)	13,594 (24.3)	
	ICU, same hospital	435 (0.2)	89 (0.2)	121 (0.2)	117 (0.2)	108 (0.2)	
	Direct ICU admission	951 (0.4)	146 (0.3)	255 (0.3)	337 (0.5)	213 (0.4)	
Hospital classification							0.697
	Metropolitan	51,856 (21.1)	11,064 (22.1)	13,721 (18.6)	16,212 (24.6)	10,859 (19.4)	
	Private	34,926 (14.2)	3,213 (6.4)	6,910 (9.3)	11,770 (17.8)	13,033 (23.3)	
	Rural/Regional	52,320 (21.3)	17,590 (35.2)	28,143 (38.1)	5,884 (8.9)	703 (1.3)	
	Tertiary	106,765 (43.4)	18,144 (36.3)	25,139 (34.0)	32,130 (48.7)	31,352 (56.0)	
APACHE 3 score		52.00 [36.00 - 69.00]	52.00 [37.00 - 70.00]	50.00 [35.00 - 68.00]	52.00 [37.00 - 70.00]	53.00 [37.00 - 70.00]	0.05
ANZROD		0.04 [0.01 - 0.13]	0.04 [0.01 - 0.14]	0.04 [0.01 - 0.13]	0.04 [0.01 - 0.13]	0.04 [0.01 - 0.13]	0.023
Interventions							
	ECMO	441 (0.2)	59 (0.1)	98 (0.1)	142 (0.2)	142 (0.3)	0.019
	Invasive ventilation	57,494 (23.4)	11,695 (23.4)	15,924 (21.5)	16,455 (24.9)	13,420 (24.0)	0.043
	CRRT	8,408 (3.4)	1,691 (3.4)	2,338 (3.2)	2,293 (3.5)	2,086 (3.7)	0.016
	Inotrope or vasopressors	55,254 (22.5)	10,875 (21.7)	15,525 (21.0)	15,516 (23.5)	13,338 (23.8)	0.041
Diagnostic categories							0.072
	Cardiac surgery	8,050 (3.5)	1,596 (3.4)	2,256 (3.3)	2,135 (3.5)	2,063 (3.9)	
	Cardiovascular	32,278 (14.0)	6,232 (13.3)	9,972 (14.4)	8,832 (14.3)	7,242 (13.9)	
	Gastrointestinal	35,778 (15.6)	7,509 (16.1)	10,730 (15.5)	9,690 (15.7)	7,849 (15.0)	
	Metabolic	27,253 (11.9)	5,423 (11.6)	8,351 (12.1)	7,238 (11.8)	6,241 (11.9)	
	Neuro	24,808 (10.8)	4451 (9.5)	7,065 (10.2)	6,660 (10.8)	6,632 (12.7)	
	Other	4,374 (1.9)	877 (1.9)	1,371 (2.0)	1,163 (1.9)	963 (1.8)	
	Renal	55,43 (2.4)	1,130 (2.4)	1,658 (2.4)	1,539 (2.5)	1,216 (2.3)	
	Respiratory	45,054 (19.6)	9,753 (20.9)	13,879 (20.0)	11,683 (19.0)	9,739 (18.6)	
	Sepsis	29,586 (12.9)	6,218 (13.3)	8,741 (12.6)	8,063 (13.1)	6,564 (12.6)	
	Trauma	17,115 (7.4)	3,546 (7.6)	5,246 (7.6)	4,546 (7.4)	3,777 (7.2)	

IRSAD - Index of Relative Socio-Economic Advantage and Disadvantage; ICU - intensive care unit; CCU - coronary care unit; HDU - high dependency unit; OT, Operating Theatre; APACHE - Acute Physiology and Chronic Health Evaluation; ANZROD - Australian and New Zealand Risk of Death; ECMO - extracorporeal membrane oxygenation; CRRT - continuous renal replacement therapy. Data are presented as n (%) or median [interquartile range] unless otherwise stated. Between-group differences are summarised with the standardized mean difference.

Compared to the top IRSAD quartile (9-10), patients in the most disadvantaged IRSAD quartile (1 - 3) are less likely to be admitted to private hospitals and tertiary hospitals. There was no significant difference in their demographic profile, ICU interventions received, or reasons for admission. Other patient characteristics, including intervention details, are available in table 1.

### Outcomes

**Figure 2:**
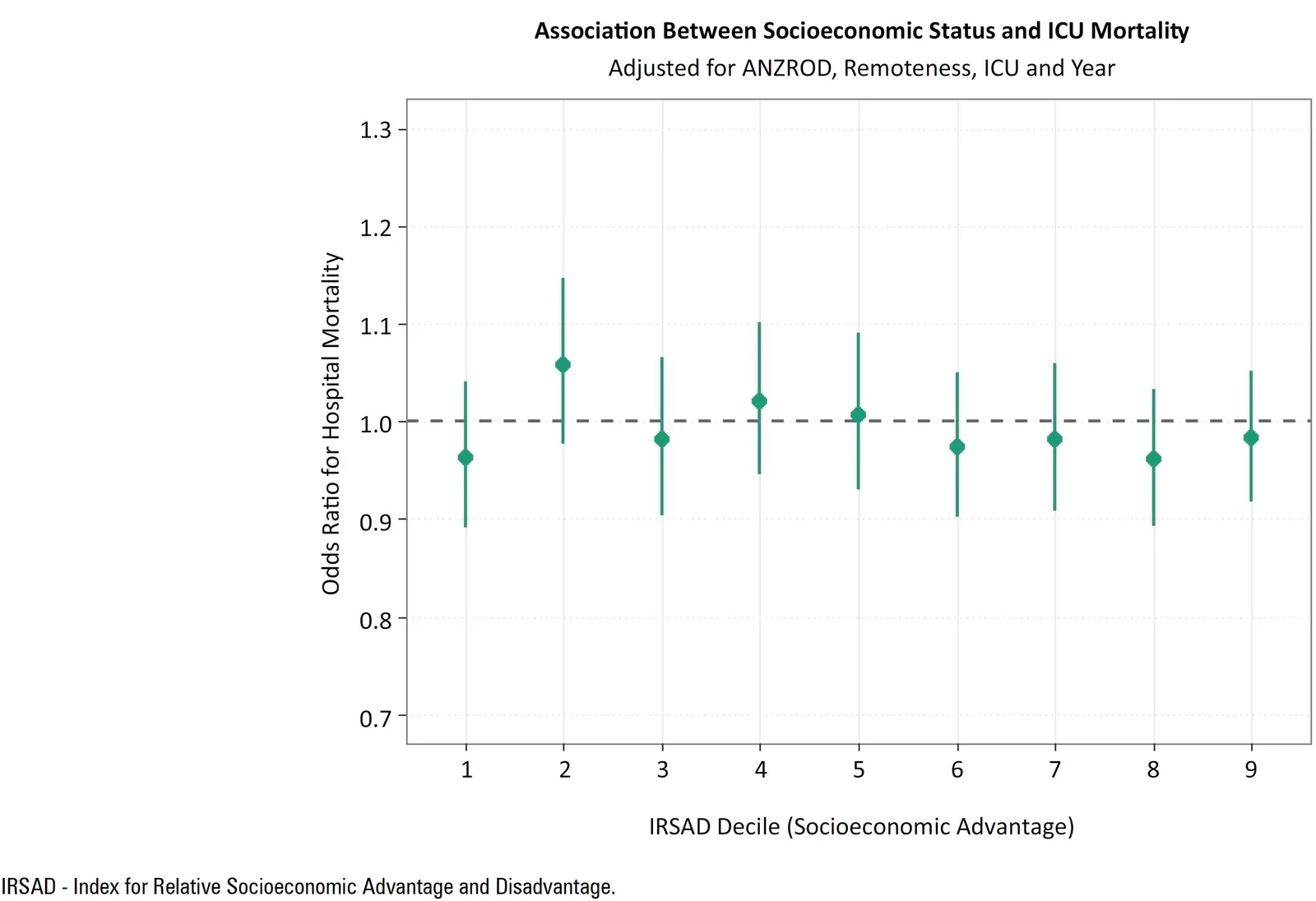
Association between Socioeconomic Status and Mortality.

Overall, unadjusted hospital mortality was 11.1%, and was similar between the lowest and highest IRSAD quartiles (Table 1S - Supplementary Material). After adjustment for severity of illness, year of admission, remoteness index, and site (as a random effect), there was no association between IRSAD and the risk of in-hospital mortality (X^2^ = 10.7, p = 0.3) (Figure 2).

Higher is more advantageous. Reference = Decile 10.

Unadjusted ICU mortality was 7.2%. The median ICU length of stay was 47.7 hours (interquartile range [IQR] 24.3 - 92.4 hours), and the median hospital length of stay was 8.07 (4.11, 15.88). Compared to the highest IRSAD Quartile, the lowest quartile had similar outcomes. Post-hospitalization destinations were similar across IRSAD quartiles, except for discharges to rehabilitation services (3.8% in the lower IRSAD quartile, compared to 6.9% in the higher IRSAD quartiles) (Table 1S - Supplementary Material).

Post-hospitalization destination was broadly similar across SES groups, although patients from more disadvantaged quartiles were less frequently discharged to inpatient rehabilitation services (Table 1S - Supplementary Material).

## DISCUSSION

In this national, multicentre study, we found that ICU patients from lower-SES backgrounds are more likely to be admitted from metropolitan or rural and regional hospitals and less likely to be discharged to rehabilitation services. All SES groups have otherwise similar demographic and clinical profiles. After multivariate risk adjustment, being from a lower SES status was not associated with risk of hospital mortality after unplanned ICU admission.

### Comparison with existing literature

Our findings contrast with a body of international literature that has identified a relationship between various SES measures and short-term ICU outcomes. Measures that have been demonstrated to be associated with ICU mortality include income,^(17)^ education^(18)^ and the degree of deprivation.^(6,19)^

Several factors may account for the differences in this study. Firstly, the methods of defining SES have varied considerably across these studies, using either single measures such as education or income, or depth of deprivation (in contrast to the IRSAD, which also accounts for the degree of advantage). Secondly, we made use of a risk adjustment approach that included methods designed specifically for our cohort.^(14)^ Finally, variations in health system design and delivery may alter the impact of SES on ICU outcomes.

Within the Australian context, evidence examining the relationship between SES and ICU outcomes has been limited. Two studies have examined the impact of SES on ICU patient outcomes, both limited to single jurisdictions but yielding similar findings. Ho examined single-center ICU patient outcomes from Western Australia and identified that the degree of deprivation was not associated with in-hospital mortality.^(12)^ More recently, Mullany did not find any association between the same SES measure used in our study and in-hospital mortality for non-elective ICU patients in Queensland, after adjustment for illness severity.^(11)^

### Implications

Our findings highlight the impact of SES on the epidemiology of unplanned ICU presentations across Australia, particularly in relation to the type of hospital in which they receive care. Patients from lower SES were more likely to be admitted to metropolitan and rural hospitals, whereas no significant differences were observed in other baseline characteristics or ICU interventions. This suggests that, amongst unplanned ICU admissions, SES is not associated with differences in presentation or management.

Importantly, we found that risk-adjusted hospital outcomes after unplanned ICU admission are not associated with SES. Whilst our study cannot exclude an upstream impact of SES on pre-ICU patient pathways, our findings indicate that the quality of care delivered to patients from the point of contact with emergency critical care services is unaffected by socioeconomic disadvantage, a finding that is particularly topical given the challenge of addressing equity in healthcare delivery.^(20)^

This finding may reflect features of the Australian healthcare system, where universal access to emergency healthcare (including ICU) is free for all eligible patients, ensuring that patients receive clinical care regardless of their ability to pay, as well as robust resourcing and access to highly qualified clinical staff, ensuring a high quality of critical care available even in rural and regional settings.^(21,22)^ However, this study was restricted to unplanned ICU admissions and cannot assess upstream pathways to ICU admissions that may be influenced by SES (e.g., access to high-risk elective surgical procedures). Similarly, the higher proportion of patients discharged to rehabilitation services amongst high SES groups may reflect differences in downstream post-acute care access, potentially due to the mixed private-public nature of rehabilitation services in Australia.

### Strengths

This study has several strengths. Firstly, we made use of a large, high-quality clinical registry that is in routine use across Australia. This provided enough power to identify differences in outcomes and characteristics between different SES groups. This produces results with greater generalisability in the Australian setting.

Secondly, we used adjustment factors that have been well-validated in the Australian setting. In particular, this study used ANZROD for risk adjustment, which has been shown to have better calibration to the Australian population than the illness scores used in other studies.^(14)^ The use of the 2016 IRSAD also ensured that the most up-to-date version of population data from the 2016 Census was used for the assessment of SES.

Thirdly, we made use of robust statistical methods to accurately assess adjusted mortality. The mixed-effects modelling allowed us to specify variables to be included as fixed effects, as is usually done in multivariate regression, whilst allowing for clustering by individual ICUs. These techniques have been argued to produce a more accurate assessment of risk-adjusted mortality than traditional epidemiological methods such as standardized mortality ratios.^(23)^

### Limitations

Firstly, this was a retrospective observational study; as such, the patterns observed cannot be assigned direct mechanistic relationships. This is less likely to be an issue in our study, where robust risk adjustment was performed. Secondly, our chosen measure of SES is assigned at the postcode level rather than the individual patient level and averages various SES indices within a postcode. Certain postcodes may experience significant heterogeneity in their SES distribution, and this ecological measure of IRSAD may not accurately capture the major socio-economic disadvantage experienced by an individual patient. Despite this, we believe the IRSAD remains a valid representation of the overall SES of a large dataset such as ours. In particular, the use of composite SES measures that average advantages and disadvantages across geographical boundaries, such as postal codes, has been shown to correlate with other health outcomes.^(24)^ Thirdly, we were unable to assess the long-term outcomes after an ICU admission, including measures such as quality of life, and further work would need to be done to examine this.

## CONCLUSION

In adult patients experiencing unplanned admissions to Australian intensive care units, there was no association between socioeconomic status and hospital mortality, suggesting that characteristics unique to the Australian healthcare setting may mitigate the adverse impact of lower socioeconomic status on intensive care unit outcomes observed in other settings.

## Data Availability

The data underlying this study were obtained from the ANZICS Adult Patient Database under a data access agreement that prohibits public release. Individual-level patient data cannot be shared due to privacy legislation (Privacy Act 1988 (Cth); Health Records Act 2001 (Vic)), ethics approval conditions, and data custodian restrictions. Aggregated summary statistics sufficient to reproduce the primary analyses are available from the corresponding author upon reasonable request, subject to execution of a data sharing agreement and ethics committee approval. The analytical code used in this study is publicly available at https://github.com/singchee/ANZICS-SES.
