# Supplementary material for: The impact of socio-economic status on outcomes after unplanned intensive care unit admissions in Australia: a retrospective observational cohort study
Source: Crit Care Sci. 2026 Jul 16;38:e20260417. doi: 10.62675/2965-2774.20260417 (PMC13518017; doi:10.62675/2965-2774.20260417)
Supplement: Supplementary file 1 [file 2965-2774-ccsci-38-e20260417-Suppl01.pdf]

Critical Care Science
https://doi.org/10.62675/2965-2774.20260417

SUPPLEMENTARY MATERIAL

The impact of socio-economic status on outcomes after unplanned intensive care unit admissions in Australia: a retrospective observational cohort study

Sing Chee Tan[1], Daniel Capurro[2], David Pilcher[3]

Table 1S - Unadjusted patient outcomes

	Overall	IRSAD quartiles			
		1 - 3	4 - 5	6 - 8	9 - 10
ICU mortality	17,225 (7.0)	3,603 (7.2)	4,846 (6.6)	4,747 (7.2)	4,029 (7.2)
Hospital mortality	27,342 (11.1)	5,608 (11.2)	7,715 (10.4)	7,539 (11.4)	6,480 (11.6)
ICU LOS (hours)	47.67 [24.32 - 92.42]	49.52 [25.35 - 94.75]	47.48 [24.13 - 91.33]	46.75 [24.00 - 91.47]	47.32 [24.18 - 92.67]
Hospital LOS (days)	8.07 (4.11 - 15.88)	8.01 (4.13 - 15.25)	7.48 (3.84 - 14.82)	8.18 (4.17 - 16.23)	8.88 (4.52 - 17.38)
Post-hospitalization destination					
Died	27,342 (11.12)	5,608 (11.21)	7,715 (10.44)	7,539 (11.42)	6,480 (11.58)
Home	166,584 (67.75)	33,025 (66.04)	50,271 (68.01)	44,881 (68.01)	38,407 (68.65)
Nursing home/chronic care/palliative care	10,020 (4.08)	1,840 (3.68)	2,935 (3.97)	2,833 (4.29)	2,412 (4.31)
Rehabilitation	12,232 (4.98)	1,906 (3.81)	2,624 (3.55)	3,871 (5.87)	3,831 (6.85)
Mental health	1,462 (0.59)	270 (0.54)	403 (0.55)	492 (0.75)	297 (0.53)
Hospital in the home	1,304 (0.53)	325 (0.65)	380 (0.51)	393 (0.60)	206 (0.37)
Other hospital/other	26,923 (10.95)	7,037 (14.07)	9,585 (12.96)	5,987 (9.07)	4,314 (7.71)

IRSAD - Index of Relative Socio-Economic Advantage and Disadvantage; ICU - intensive care unit; LOS - length of stay. Data are presented as n (%) or median [interquartile range]

This is an open access article under the CC BY license (https://creativecommons.org/licenses/by/4.0/).

Table 2S - Contributing sites

Site name
Albury Wodonga Health ICU
Alfred Hospital ICU
Alice Springs Hospital ICU
Angliss Hospital ICU
Armadale Health Service ICU
Ashford Community Hospital ICU
Austin Hospital ICU
Bankstown-Lidcombe Hospital ICU
Bathurst Base Hospital ICU
Bendigo Health Care Group ICU
Blacktown Hospital ICU
Box Hill Hospital ICU
Brisbane Private Hospital ICU
Buderim Private Hospital ICU
Bunbury Regional Hospital ICU
Bundaberg Base Hospital ICU
Caboolture Hospital ICU
Cabrini Hospital ICU
Cairns Hospital ICU
Calvary Adelaide Hospital ICU
Calvary Hospital (Lenah Valley) ICU
Calvary John James Hospital ICU
Calvary Mater Newcastle ICU
Calvary North Adelaide Hospital ICU
Campbelltown Hospital ICU
Canberra Hospital ICU
Central Gippsland Health Service (Sale) ICU
Coffs Harbour Health Campus ICU
Concord Hospital (Sydney) ICU
Dandenong Hospital ICU
Dubbo Base Hospital ICU
Epworth Eastern Private Hospital ICU
Epworth Freemasons Hospital ICU
Epworth Geelong ICU
Epworth Hospital (Richmond) ICU
Fairfield Hospital ICU
Fiona Stanley Hospital ICU
Flinders Medical Centre ICU
Flinders Private Hospital ICU
Footscray Hospital ICU
Frankston Hospital ICU
Gold Coast Private Hospital ICU
Gold Coast University Hospital ICU
Gosford Hospital ICU
Gosford Private Hospital ICU
Goulburn Base Hospital ICU
Goulburn Valley Health ICU
Grafton Base Hospital ICU
Grampians Health Ballarat ICU
Grampians Health Horsham ICU
Greenslopes Private Hospital ICU
Griffith Base Hospital ICU
Hervey Bay Hospital ICU
Hollywood Private Hospital ICU
Holmesglen Private Hospital ICU
Hornsby Ku-ring-gai Hospital ICU
Hurstville Private Hospital ICU
Ipswich Hospital ICU
John Fawkner Hospital ICU
John Flynn Private Hospital ICU
John Hunter Hospital ICU
Joondalup Health Campus ICU
Kareena Private Hospital ICU
Knox Private Hospital ICU
Latrobe Regional Hospital ICU
Launceston General Hospital ICU
Lingard Private Hospital ICU
Lismore Base Hospital ICU
Liverpool Hospital ICU
Logan Hospital ICU
Lyell McEwin Hospital ICU
Mackay Base Hospital ICU
Macquarie University Private Hospital ICU
Maitland Hospital ICU
Maitland Private Hospital ICU
Manly Hospital & Community Health ICU
Manning Rural Referral Hospital ICU
Maroondah Hospital ICU
Mater Adults Hospital (Brisbane) ICU
Mater Private Hospital (Brisbane) ICU
Mater Private Hospital (Sydney) ICU
Mater Private Hospital (Townsville) ICU
Melbourne Private Hospital ICU
Mildura Base Public Hospital ICU

Monash Medical Centre-Clayton Campus ICU
Mount Hospital ICU
Mount Isa Hospital ICU
Mulgrave Private Hospital ICU
National Capital Private Hospital ICU
Nepean Hospital ICU
Newcastle Private Hospital ICU
Noosa Hospital ICU
North Canberra Hospital ICU
North Shore Private Hospital ICU
North West Regional Hospital (Burnie) ICU
Northeast Health Wangaratta ICU
Northern Beaches Hospital ICU
Norwest Private Hospital ICU
Orange Base Hospital ICU
Peninsula Private Hospital ICU
Pindara Private Hospital ICU
Port Macquarie Base Hospital ICU
Prince of Wales Hospital (Sydney) ICU
Prince of Wales Private Hospital (Sydney) ICU
Princess Alexandra Hospital ICU
Queen Elizabeth II Jubilee Hospital ICU
Redcliffe Hospital ICU
Robina Hospital ICU
Rockhampton Hospital ICU
Rockingham General Hospital ICU
Royal Adelaide Hospital ICU
Royal Brisbane and Women's Hospital ICU
Royal Darwin Hospital ICU
Royal Melbourne Hospital ICU
Royal North Shore Hospital ICU
Royal Perth Hospital ICU
Royal Prince Alfred Hospital ICU
Shoalhaven Hospital ICU
Sir Charles Gairdner Hospital ICU
South West Healthcare (Warrnambool) ICU
St Andrew's Hospital (Adelaide) ICU
St Andrew's Hospital Toowoomba ICU
St Andrew's Private Hospital (Ipswich) ICU
St Andrew's War Memorial Hospital ICU
St George Hospital (Sydney) ICU
St George Private Hospital (Sydney) ICU
St John Of God Health Care (Subiaco) ICU
St John of God Hospital (Bendigo) ICU
St John of God Hospital (Berwick) ICU
St John Of God Hospital (Geelong) ICU
St John Of God Hospital (Murdoch) ICU
St John of God Midland Public & Private ICU
St Vincent's Private Hospital Northside ICU
St Vincent's Hospital (Sydney) ICU
St Vincent's Hospital (Toowoomba) ICU
St Vincent's Private Hospital (Sydney) ICU
St Vincent's Private Hospital Fitzroy ICU
Sunnybank Hospital ICU
Sunshine Coast University Hospital ICU
Sunshine Coast University Private Hospital ICU
Sunshine Hospital ICU
Sutherland Hospital & Community Health Services ICU
Sydney Adventist Hospital ICU
Sydney Southwest Private Hospital ICU
Tamworth Base Hospital ICU
The Bays Hospital ICU
The Chris O'Brien Lifehouse ICU
The Memorial Hospital (Adelaide) ICU
The Northern Hospital ICU
The Prince Charles Hospital ICU
The Queen Elizabeth (Adelaide) ICU
The Wesley Hospital ICU
Toowoomba Hospital ICU
Townsville University Hospital ICU
Tweed Valley Hospital ICU
University Hospital Geelong ICU
Wagga Wagga Base Hospital & District Health ICU
Warringal Private Hospital ICU
Werribee Mercy Hospital ICU
Western District Health Service (Hamilton) ICU
Western Hospital (SA) ICU
Western Private Hospital ICU
Westmead Hospital ICU
Westmead Private Hospital ICU
Wollongong Hospital ICU
Wollongong Private Hospital ICU
Women's and Children's Hospital Pediatric ICU
Wyong Hospital ICU

ICU - intensive care unit.
